# TDP-SAR: Task-Driven Pruning Method for Synthetic Aperture Radar Target Recognition Convolutional Neural Network Model

**DOI:** 10.3390/s25103117

**Published:** 2025-05-15

**Authors:** Tong Zheng, Qing Wu, Chongchong Yu

**Affiliations:** 1 School of Computer and Artificial Intelligence, Beijing Technology and Business University, Beijing 100048, China; chongzhy@vip.sina.com; 2 Heilongjiang Province Key Laboratory of Laser Spectroscopy Technology and Application, Harbin University of Science and Technology, Harbin 150006, China; wuqing@buaa.edu.cn

**Keywords:** synthetic aperture radar (SAR), SAR target recognition, speckle noise, pruning, spectrum analysis

## Abstract

Synthetic aperture radar (SAR) target recognition plays a crucial role in SAR image interpretation. While deep learning has become the predominant approach for SAR target recognition, existing methods face practical deployment challenges due to excessive model complexity. In addition, SAR images are less understandable compared to optical images, which leads to greater difficulties in analyzing the target features of SAR images in the spatial domain. To address the above limitation, we propose a novel task-driven pruning (TDP-SAR) strategy. Unlike conventional pruning techniques that rely on generic parameter importance metrics, our approach implements frequency domain analysis of convolutional kernels across different processing stages of SAR target recognition models. In the experimental section, we use the MSTAR benchmark dataset to prove that the TDP-SAR can not only effectively compress the model size but also adapt to different quality SAR images compared to baseline architectures. Particularly when processing the joint convolutional neural network (J-CNN) model proposed in the previous study, the number of parameters decreased by 17.7% before and after pruning. This advancement facilitates the practical deployment of deep learning solutions in resource-constrained SAR interpretation systems while preserving recognition reliability.

## 1. Introduction

Synthetic aperture radar (SAR) is known as an active microwave imaging system using coherent signal processing to achieve high-resolution terrain observation and the synthesis of a virtual, large-aperture antenna from the relative motion between the radar platform and targets [1,2]. Notably, SAR exhibits unique penetration characteristics, allowing microwave signals to interact with subsurface layers through cloud cover, vegetation canopies, and certain man-made obstacles. These are capabilities unattainable in optical imaging systems. Current SAR applications predominantly focus on raw image generation, yet critical challenges persist in automated target interpretation. The effective utilization of SAR data requires a systematic extraction of discriminative target features from complex scattering signatures, followed by an accurate characterization of physical properties through advanced image interpretation techniques. This paper specifically addresses the technical bottlenecks in SAR target recognition, with emphasis on robust feature representation and reliable information extraction under operational constraints.

Deep learning-based SAR image target recognition methods use computational systems to automatically extract deeper, more detailed, and more abstract feature representations, whereas they use traditional target recognition algorithms that rely on human-designed features with relatively simplistic attribute characteristics. Furthermore, in the meantime, deep learning frameworks use end-to-end neural network (NN) structures that combine both processes by means of unified optimization, whereas traditional approaches usually divide feature extraction and classification into discrete stages. Deep learning-based SAR target recognition approaches have been used in the last several years to become the main topic of SAR target identification [3].

Chen et al., from Fudan University, developed an all-convolutional neural network architecture (A-ConvNets) [4]. By eliminating the fully connected layers in conventional convolutional neural network (CNN) models, this design significantly reduces the number of trainable parameters, thereby mitigating overfitting issues inherent in conventional architectures. Addressing this challenge, Fu et al. proposed a random deactivation mechanism integrated into deep residual networks, combining the center loss function with the Softmax classifier for joint optimization [5]. To enhance model robustness, Ding et al. at Xidian University implemented data augmentation techniques, including rotation and translation, to diversify training samples [6]. In [7], a generative adversarial network (GAN) was used to construct realistic SAR imagery by adversarial training between generator and discriminator modules, effectively increasing datasets for SAR target recognition. Building upon this framework, Ledig et al. enhanced GAN performance by adding a geometric feature-aware loss function, achieving increased recognition accuracy [8]. Reference [9] applies regularization strategies to decrease noise-induced feature variations, thereby decreasing speckle noise interference in SAR target recognition. In [10], a capsule network architecture is proposed to preserve pose-encoded features while keeping intrinsic spatial links, demonstrating better performance in SAR applications. Building on this, Ren et al. created a dilated convolutional capsule network that uses hierarchical capsule units to extract multi-scale features, greatly increasing the robustness of recognition by explicitly modeling feature spatial dependencies [11]. In order to make the most of prominent visual patterns from SAR imagery easier to extract and to increase computational efficiency and classification accuracy, the integration of attention pathways into CNN architectures is studied in [12]. Interestingly, although deep learning and conventional approaches have different advantages, current research directions highlight hybrid frameworks that work in concert with deep neural representations and handcrafted feature engineering. This paradigm leverages complementary strengths of both methodologies, positioning itself as a pivotal developmental trajectory in SAR target recognition technology. Reference [13] proposed a Gabor transform-based CNN architecture using multi-scale, multidirectional Gabor filters to construct enriched training datasets, thereby increasing data variety and reducing overfitting. In [14], rotation-invariant multi-scale features (encoding local texture and edge semantics) are combined with CNN-derived representations, achieving hierarchical feature representations for SAR target recognition. Reference [15] introduced a CNN-driven dictionary learning framework, employing ConvNet as an automated feature extractor and building a discriminative dictionary for sparse sample representation. With the use of traditional dictionary learning, this method is able to overcome the shortcomings of being dependent on the use of handcrafted feature engineering. As explained in [16], the research group used nonlinear mapping to project CNN features into a reproducing kernel Hilbert space for improved separability in order to address nonlinear dictionary training dynamics by broadening this work. Reference [17] proposes a CNN model that can process covariance and polarimetric coherence matrices, which will allow for the extraction of spatial features with hierarchical polarization. The use of deep learning in polarimetric SAR target recognition is demonstrated in this paper, which also shows its potential for use in the past. Pioneering feature fusion research, Zhang et al. developed a hybrid framework integrating scattering center attributes with CNN-derived representations. Their methodology applies discriminative correlation analysis (DCA) to enhance feature complementarity, significantly boosting SAR recognition robustness [18]. However, this approach inevitably compromises the geometric integrity of feature maps through the vectorization of spatial hierarchies. Addressing this limitation, the research team implemented adaptive fusion within 2D feature embedding spaces, preserving geometric–semantic relationships while optimizing cross-modal feature synergy. This advancement establishes new benchmarks for scattering–CNN feature fusion efficacy [19]. In contrast to optical imagery, SAR images exhibit inherent speckle noise that severely degrades visual quality and compromises downstream target recognition accuracy. To address this dual challenge, we propose a novel joint convolutional neural network (J-CNN) with dual-task architecture, enabling simultaneous speckle suppression and target classification through end-to-end optimization. This framework demonstrates robust adaptability across SAR datasets with heterogeneous noise levels, establishing a unified solution for enhanced recognition reliability [20].

The development of deep neural networks was studied; as the number of network layers increased, so did the corresponding gains in recognition accuracy. However, this architectural growth has a quadratic increase in parameter volume, which has resulted in a corresponding increase in the amount of storage resources that significantly restricts the use of deep models in practice. Model compression techniques, such as pruning, quantization, and knowledge distillation, as well as low-rank decomposition, have become important research areas to lessen the bottleneck. Model pruning in this paradigm uses structural saliency assessments, which are quantified by metrics including parameter magnitude, weight density, and convolutional kernel entropy, as well as redundant components. This process achieves simultaneous reduction in both parameter count and computational load, while post-pruning accuracy is recovered through systematic fine-tuning. The conceptual foundation of model pruning was established as early as 1989, when LeCun introduced a parameter saliency criterion based on loss function Hessian analysis, enabling connection reduction through second-order optimization principles [21]. This pioneering work laid the theoretical groundwork for subsequent pruning research paradigms. Significantly advancing the field, Han et al. developed deep compression, a unified framework synergistically integrating pruning, quantization, and Huffman coding [22]. Through repeated magnitude-based thresholding and treatment parameters as statistically independent entities, their approach used non-structured pruning, with sparsity-aware retraining maintaining baseline accuracy. Using these fundamentals, Molchanov et al. created the idea of pruning as a combinatorial optimization problem and used gradient-informed importance scoring to find low-cost parameter subsets that maintained model fidelity after pruning [23]. But because of the way that these methods are composed of unstructured pruning approaches, which cause neural architectures to have irregular connection patterns, sparsity constraints are enforced to reduce memory footprints. Such structural irregularities call for the explicit indexing mechanisms for non-zero element identification during the inference phase, which naturally makes the computational network incompatible with parallel computation architectures. Furthermore, the practical use of sparse models requires the need for specific hardware acceleration libraries to effectively take advantage of the sparsity-aware computation primitives.

Recent advances in pruning methodology have prioritized structured pruning paradigms, where convolutional weight elimination is governed by regularization-driven structural constraints. For instance, Wen et al. implement group Lasso regularization to enforce group-wise sparsity, systematically driving parameter clusters towards numerical insignificance [24]. Similarly, Jin et al. introduce an L0 norm penalty approximated by iterative reparameterization, though this regularization augmentation incurs considerable convergence overhead [25]. Notably, coarse-grained pruning methods, spanning convolutional filter removal, input channel truncation, and feature map suppression, demonstrate layer-wise interdependency. Namely, pruning the *i*-th layer’s filters necessitates a proportional elimination of dependent feature maps and subsequent (*i* + 1)-th layer kernels, thereby maintaining dimensional compatibility across network hierarchies.

Current pruning methodologies exhibit task-agnostic limitations, failing to incorporate domain-specific prior knowledge. This deficiency becomes critically pronounced in SAR image processing, where spatial domain variance, stemming from radar parameter sensitivity, introduces non-stationary input distributions that compromise model input consistency. Paradoxically, data-driven pruning frameworks demonstrate pathological fragility to SAR domain shifts, thereby casting doubt on the operational integrity of pruned architectures in real-world reconnaissance scenarios. To address this dual challenge, we propose a task-oriented pruning framework that explicitly optimizes parameter saliency against mission-critical objectives. Our methodology strategically eliminates task-irrelevant parameters through the joint optimization of model sparsity and target recognition fidelity. At the same time, the stability and reliability of the model can be ensured as much as possible. Our work advances the practical deployment of deep learning models for SAR target recognition.

The remainder of our paper is structured as follows: Section 2 describes related studies on SAR images, including a classical CNN model used for SAR target recognition, speckle noise characteristic of SAR images, and the J-CNN model structure. Then, the strategy of the proposed TDP-SAR is outlined in Section 3. In Section 4, we provide experiments to verify the effectiveness of TDP-SAR. Finally, the research content of this paper is summarized in Section 5.

## 2. Related Works

### 2.1. Classical CNN Model Used for SAR Target Recognition

Commonly used CNN architectures such as AlexNet [26], visual geometry group (VGG) [27], and residual networks (ResNet) [28] were implemented for SAR target recognition. To ensure classification performance, these models were trained and fine-tuned using the public MSTAR dataset. Through parameter optimization, the pre-trained CNN models achieved an effective classification of 10 military target categories. The structure characteristics of these models are as follows:

(1) AlexNet

Five convolutional layers make up the AlexNet architecture, which consists of three pooling and three fully connected layers. It created deeper convolutional layers and a much bigger receptive field especially designed to handle large-scale datasets such as ImageNet in contrast to previous CNN models. AlexNet was founded as a transitional architecture in computer vision research by this groundbreaking architecture, which is used to bridge both deep and shallow neural networks.

(2) VGG

In contrast to AlexNet’s architecture, the VGG network uses smaller convolutional kernels (3 × 3) to increase computational efficiency. To keep sufficient receptive field dimensions, successively deeper variants (VGG11, VGG13, VGG16, and VGG19) were developed by layered stacking, as systematically categorized in [27]. For controlled comparison analysis in our trials, VGG16 works as the baseline architecture.

(3) ResNet

ResNet strategically use batch normalization (BN) layers to solve gradient vanishing/explosion problems in deep networks. By using residual connections that create skip linkages between non-adjacent layers, this architecture effectively decouples layer dependencies while keeping feature map integrity. These improvements jointly alleviate network degradation, permitting good training of 18- to 152-layer variants (ResNet-18/34/50/101/152). For methodological consistency, our experiments especially chose ResNet-50 as the representative architecture.

### 2.2. Speckle Noise of SAR Images

Every resolution cell includes many different types of distinct scatterers inside the SAR image structure, which are based on a given area in which there will be many different kinds of scatterers in the area. The result that is obtained from the uninterrupted superposition of individual scatterer responses is the echo vector, involving the main limit theorem [29]; in the deep learning model that is being developed, the artificial radar echo formulation is modeled in a complicated Gaussian way:

(1) $$Ae^{j\phi }=z_{1}+jz_{2},$$

where A and ϕ are the amplitude and phase of the signal, respectively. $z_{1}$ and $z_{2}$ are the real and imaginary parts. The complex signal components exhibit a circular system, with both real and imaginary parts following independent identically distributed (i.i.d.) Gaussian processes [30], namely $ℜ\left(s\right)∼N\left(0,\sigma^{2}\right)$ and $ℑ\left(s\right)∼N\left(0,\sigma^{2}\right)$, where $\sigma^{2}$ denotes the common variance parameter. Hence, the joint probability distribution function (PDF) can be expressed as

(2) $$p_{z_{1},z_{2}}\left(z_{1},z_{2}\right)=\frac{1}{\pi \sigma }e^{-\frac{z_{1}^{2}+z_{2}^{2}}{\sigma }},$$

where σ denotes the standard deviation. Furthermore, the intensity follows an exponential distribution, and the corresponding PDF is

(3) $$p_{I}\left(I_{n}\right)=\frac{I_{n}}{\sigma }e^{-\frac{I_{n}}{\sigma }},$$

where *I_n_* is the intensity. In the SAR system, an observation sample is usually measured independently for *L* times. Incoherent average processing is carried out to obtain the final measurement result. In order to obtain the PDF of the observed intensity, Equation (3) needs to be convolved *L* times. The result is

(4) $$p_{\left.I_{L}\right|\sigma }\left(\left.I_{L}\right|\sigma \right)=\frac{1}{\Gamma \left(L\right)}{\left(\frac{L}{\sigma }\right)}^{L}I_{L}^{L-1}e^{-\frac{LI_{L}}{\sigma }},$$

where the observed intensity follows the gamma distribution, whose shape parameters α and scale parameters β are *L* and L/σ, respectively. Under the fully developed speckle assumption [31,32], SAR intensity observations exhibit strict adherence to a multiplicative noise model when the sampling interval matches the system’s spatial resolution, expressed mathematically as

(5) $$I_{unit}=R_{t}\cdot u,$$

where *R_t_* includes backscattering characteristics. *u* is multiplicative speckle noise independent of *R_t_*. The speckle component in fully developed conditions obeys a Gamma distribution parametrized as $u∼\Gamma \left(L,1/L\right)$, exhibiting a unit mean ($E\left(u\right)=1$) and variance inversely proportional to the equivalent number of looks ($Var\left(u\right)=1/L$). In other words, α=β=L. Then, we substitute σ=1 into Equation (4), and we can find that

(6) $$p\left(u\right)=\frac{L^{L}\cdot u^{L-1}}{\Gamma \left(L\right)}e^{-Lu},$$

**Figure 1:**
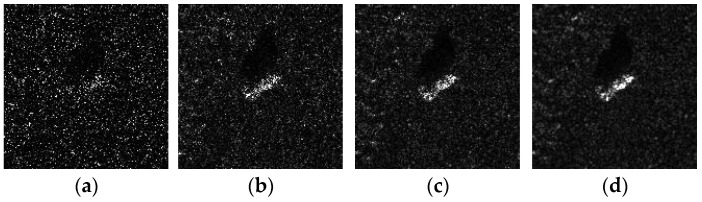
Comparison of the SAR images of the same BMP2 target with different levels of speckled noise: (**a**) *L* = 0.2; (**b**) *L* = 1; (**c**) *L* = 5; (**d**) original SAR image.

As can be seen from Equation (5), the sharper the gray changes in the image, the faster the noise varies. To empirically validate this relationship, we conduct controlled experiments using BMP2 target chips from the MSTAR dataset. Synthetic speckle contamination is implemented with varying equivalent look numbers *L*, producing the comparative intensity images shown in Figure 1.

### 2.3. J-CNN Model Used for SAR Target Recognition

**Figure 2:**
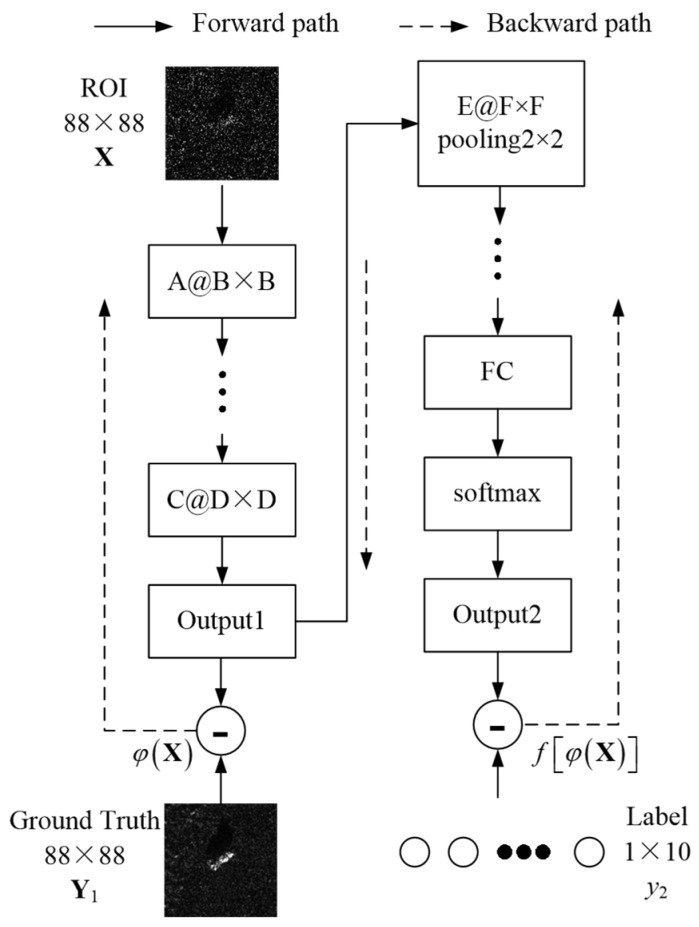
Overall structure of J-CNN.

The J-CNN architecture proposed in Reference [20] addresses speckle-induced recognition degradation through synergistic despeckling–recognition integration, whose structure is shown in Figure 2. The architectural foundation of this framework lies in the synergistic integration between the despeckling module and recognition module. Through two-stage complementary feature processing, the robustness of the SAR target recognition model is improved. The J-CNN consists of convolutional layers, pooling layers, one full connection (FC) layer, and one softmax classifier. Concretely, the SAR images after synthesizing speckle noises, X, are disposed by the despeckling process, which can improve the image quality. The despeckled SAR images, namely Output1, are expressed by $\varphi \left(X\right)$. Then, the recognition results can be obtained by the recognition process, namely Output2, as $f\left[\varphi \left(X\right)\right]$. During model optimization, the stochastic gradient descent (SGD) framework synchronously processes dual-task objectives: the despeckling module computes reconstruction errors, while the recognition module evaluates classification discrepancies. These coordinated gradients collectively drive parameter updates through backpropagation. The unified backpropagation framework exhibits asymmetric gradient propagation characteristics. Recognition module parameters are governed by task-specific gradients excluding regularization terms, whereas the despeckling module updates incorporate interdependent gradients from both reconstruction and discriminative objectives. In view of this, the total loss function is designed as:

(7) $$L\left(w\right)={\left\|f\left[\varphi \left(X\right)\right]-y_{2}\right\|}_{2}+\lambda \cdot {\left\|\varphi \left(X\right)-Y_{1}\right\|}_{2}+\eta \cdot {\left\|w\right\|}_{1},$$

where $Y_{1}$ comprises SAR images with high quality, $y_{2}$ is the actual target label, w are parameters of the model, and λ and η are hyperparameters used to adjust the two-stage loss function and the degree of effect of the regularization term on network parameter updating. Moreover, ||·||_1_ and ||·||_2_ represent the 1-norm and 2-norm, respectively.

Table 1 shows the systematic documentation of the convolutional layer arrangements of the pre-trained CNN model in terms of kernel size and population counts. The notation **A**@**B** × **B** represents a parameter group containing **A** individual kernels, where each kernel maintains **B** × **B** spatial resolution.

Table 2 shows the comparison between the J-CNN and classical CNN, including two aspects, namely the number of parameters and model achievements. It can be seen from Table 2 that the complexity of the classical CNN model is stronger, which is also the reason why its processing performance for tasks with a large amount of data has improved. The parameter number of the J-CNN model is significantly smaller than that of the classical CNN, which makes it suitable for SAR image datasets with relatively small data volumes and avoids overfitting of the model.

## 3. Methodology

### 3.1. Overall Strategy of TDP-SAR

**Figure 3:**
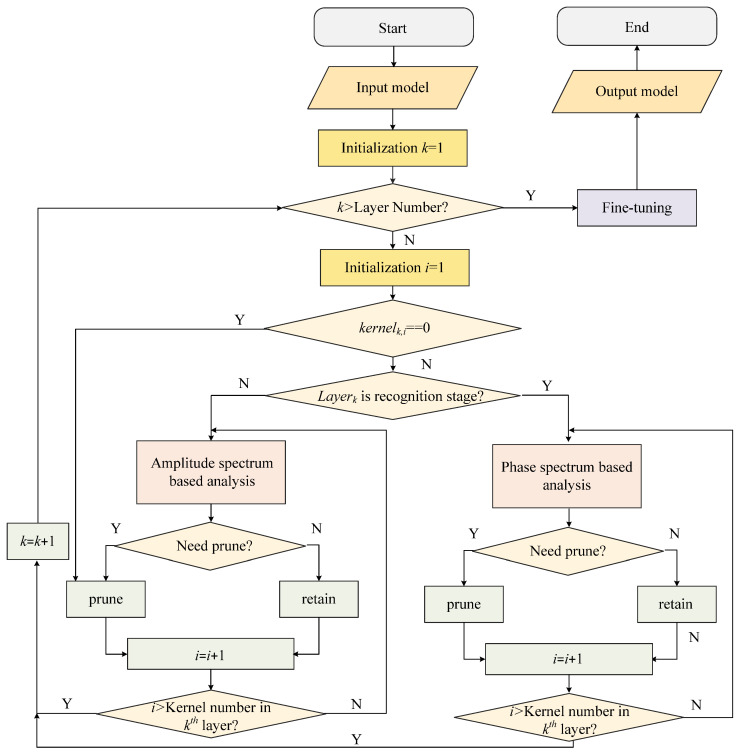
The overall process of TDP-SAR.

In the joint CNN design, there is a task-differentiated pruning mechanism that is introduced in the TDP-SAR framework. Stage-specific feature importance analysis is used to determine the pruning criteria. Recognition components concentrate on discriminative feature preservation, whereas the rest of the attention is given to spatial coherence patterns by the despeckling modules. Using the workflow visualization shown in Figure 3, this dual-task optimization approach automatically determines the kernel pruning configurations. Specifically, the model to be analyzed serves as the input for TDP-SAR, while the pruned final model constitutes the output. The procedure initiates by setting the layer index to *k* = 1. Prior to conducting convolutional kernel analysis for each layer, a conditional check is performed to verify whether the current layer index has exceeded the total number of model layers. When *k* is not more than the layer count, convolutional kernel analysis is executed; otherwise, the analysis process is terminated and proceeds directly to the fine-tuning phase. During the convolutional kernel analysis process, the kernel index of the current layer should first be initialized as *i* = 1. Subsequently, an evaluation is conducted to determine whether its magnitude values exhibit near-zero characteristics across all dimensions. If confirmed, this indicates the kernel has not contributed to the model’s functionality and can be directly pruned. For kernels demonstrating non-negligible magnitudes, a segmented rationality assessment of the workflow is performed to determine pruning eligibility. For despeckling layers, an amplitude spectrum-based assessment is conducted to determine convolutional kernel pruning eligibility, whereas for recognition layers, the evaluation is performed through phase spectrum analysis to ascertain whether the kernel requires pruning. This iterative implementation ensures all convolutional kernels across network layers are subjected to TDP-SAR pruning operations, followed by data-driven parameter refinement using training datasets, ultimately yielding optimized pruned model architecture.

### 3.2. Amplitude Spectrum Analysis

**Figure 4:**
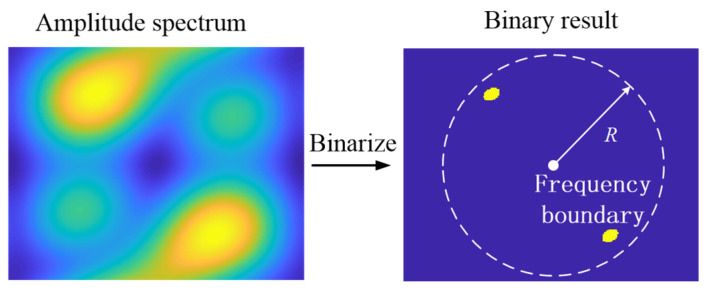
Schematic of frequency band division.

SAR imaging systems generate discrete spatial domain representations requiring spectrum transformation via 2D discrete Fourier transform (DFT). For a *P* × *Q* dimensional image configuration, this computational process converts spatial data into frequency domain components through systematic phase amplitude decomposition. The 2D-DFT result of *f*(*x*, *y*) is expressed as follows:

(8) $$F\left(u,v\right)=F\left[f\left(x,y\right)\right]=\frac{1}{N^{2}}\sum_{x=0}^{P-1}\sum_{y=0}^{Q-1}f\left(x,y\right)\cdot e^{-j2\pi \left(\frac{ux}{N}+\frac{vy}{N}\right)}=\left|F\left(u,v\right)\right|\cdot e^{j\varphi \left(u,v\right)},$$

where $F\left[\cdot \right]$ is the symbolic representation of 2D-DFT processing. $\left|F\left(u,v\right)\right|$ and $\varphi \left(u,v\right)$ are the amplitude and phase spectrum, respectively. The amplitude and phase spectra are shown as

(9) $$\left|F\left(u,v\right)\right|=\sqrt{R^{2}\left(u,v\right)+I^{2}\left(u,v\right)},$$

(10) $$\varphi \left(u,v\right)=\arctan\left[\frac{I\left(u,v\right)}{R\left(u,v\right)}\right],$$

where *R*(*u*, *v*) are *I*(*u*, *v*) real and imaginary parts of *F*(*u*, *v*), respectively. Spectrum centering is implemented to align the coordinate origin with the zero-frequency component. Frequency magnitudes demonstrate radial progression from this central reference. Under the convolution theorem framework, spatial domain convolution operations correspond to element-wise multiplication in the frequency domain. This can be shown as

(11) $$f\left(x,y\right)⊗k\left(x,y\right)\overset{F}{↔}F\left(u,v\right)K\left(u,v\right),$$

where *f*(*x*, *y*) is the SAR image; *k*(*x*, *y*) is the convolutional kernel of the CNN model; and ⊗ is the convolution processing. *F*(*u*, *v*) and *K*(*u*, *v*) are the spectra of *f*(*x*, *y*) and *k*(*x*, *y*), respectively. The feature spectrum of the convolutional layer inherently manifests as the complex product of input SAR imagery and kernel spectrum components. This fundamentally establishes the convolution theorem as the foundational framework for frequency domain operator analysis in neural processing systems. On this basis, the change in the amplitude spectrum after the convolution process can be expressed as

(12) $$\left|F\left(u,v\right)\cdot K\left(u,v\right)\right|=\left|\left|F\left(u,v\right)\right|e^{j\varphi_{1}\left(u,v\right)}\cdot \left|K\left(u,v\right)\right|e^{j\varphi_{2}\left(u,v\right)}\right|=\left|F\left(u,v\right)\right|\cdot \left|K\left(u,v\right)\right|,$$

where |*F*(*u*, *v*)| and |*K*(*u*, *v*)| are amplitude spectra of *f*(*x*, *y*) and *k*(*x*, *y*), respectively, and $\varphi_{1}\left(u,v\right)$ and $\varphi_{2}\left(u,v\right)$ are phase spectra of *f*(*x*, *y*) and *k*(*x*, *y*), respectively. Analytical derivation confirms that the convolutional amplitude spectrum obeys multiplicative composition rules between input features and kernel components. The J-CNN despeckling phase necessitates coherent noise isolation, given speckles’ characteristic manifestation in high-frequency spectrum regions. This operational paradigm motivates TDP-SAR’s implementation of kernel spectrum energy proportion metrics for despeckling efficacy quantification. If the value exceeds the threshold, $th_{r}$, the convolutional kernel in the despeckling stage is pruned. Here, $th_{r}$ can be confirmed by the Neyman–Pearson lemma. This criterion can be expressed as

(13) $$r=\frac{P_{h}}{P_{l}},$$

where *P_h_* is the point number with a value of 1 in the high-frequency band after amplitude spectrum binarization and *P_l_* is the point number with a value of 1 in the low-frequency band after amplitude spectrum binarization. Here, the division of high- and low-frequency bands is shown in Figure 4. Firstly, we binarize the amplitude spectrum and confirm the band boundary to *R*. Then, we can count the point numbers, i.e., *P_h_* and *P_l_*. Finally, the high frequency ratio can be obtained from Equation (13).

### 3.3. Phase Spectrum Analysis

According to the convolution theorem, the phase spectrum after convolution processing is expressed as

(14) $$\begin{array}{ll} r & =\frac{P_{h}}{P_{l}}\phi \left[F\left(u,v\right)\cdot K\left(u,v\right)\right]=\phi \left[\left|F\left(u,v\right)\right|e^{j\varphi_{1}\left(u,v\right)}\cdot \left|K\left(u,v\right)\right|e^{j\varphi_{2}\left(u,v\right)}\right]\\ & =\varphi_{1}\left(u,v\right)+\varphi_{2}\left(u,v\right) \end{array},$$

where $\phi \left[\cdot \right]$ is the process of obtaining the phase spectrum. Equation (14) reveals that the convolutional phase spectrum exhibits additive properties derived from input features and kernel components. Contrasting with amplitude components that encode energy distributions, the phase spectrum critically encodes structural geometry and spatial relationships. However, the raw phase spectrum exhibits limited perceptual interpretability through direct observation. To address this limitation, we developed a phase reconstruction framework within conventional SAR phase analysis, enabling the systematic identification of geometric signatures embedded in phase data. The methodology initiates with full-spectrum acquisition of raw SAR data, followed by systematic amplitude normalization through unit amplitude configuration. After 2D-IDFT, the reconstruction SAR image can be obtained, expressed by *f_re_*(*x*, *y*). The reconstruction process can be shown as

(15) $$f_{re}\left(x,y\right)=F^{-1}\left[I_{1}\left(u,v\right)\cdot e^{j\cdot \varphi \left(u,v\right)}\right],$$

where $I_{1}\left(u,v\right)$ is an all-1 matrix with a size of **A** × **B**; $F^{-1}\left[\cdot \right]$ is the 2D-IDFT process. While native SAR imagery contains both amplitude and phase spectrum components, the reconstructed imagery through inversion processes exclusively exhibits phase information retention. This selective preservation mechanism maintains the original phase characteristics while intentionally discarding amplitude modulations.

TDP-SAR employs phase correlation metrics between pre- and post-processed reconstructions to quantify geometric feature extraction efficacy through convolutional phase interactions. This quantification mechanism primarily supports target recognition protocols. The methodological workflow initiates with baseline SAR image reconstruction via spectrum inversion, followed by synthetic phase generation through the additive synthesis of original and kernel phase components. The final reconstruction, i.e., *f_r_*(*x*, *y*), implements phase-informed synthesis using unit amplitude spectrum constraints through inverse Fourier transformation. The demonstrated disparity between baseline *f_k_*(*x*, *y*) and processed reconstructions originates *f_r_*(*x*, *y*) from convolutional phase characteristics. The above process can be expressed by Equations (16) and (17). The analytical framework requires a phase correlation assessment between these reconstructed images to quantify spectrum influence, like Equation (18). This metric inversely correlates with phase operational efficacy: elevated correlation values indicate diminished phase modulation effects, corresponding to reduced geometric discriminative capacity. Conversely, lower coefficients reveal enhanced phase-driven feature extraction capabilities.

(16) $$f_{k}\left(x,y\right)=F^{-1}\left[I\left(u,v\right)\cdot e^{j\phi \left(u,v\right)}\right],$$

(17) $$f_{r}\left(x,y\right)=F^{-1}\left\{I\left(u,v\right)\cdot e^{j\left[\phi \left(u,v\right)+\phi_{k}\left(u,v\right)\right]}\right\},$$

(18) $$Cor=Corrcoef\left[f_{k}\left(x,y\right),f_{r}\left(x,y\right)\right],$$

where $\phi \left(u,v\right)$ and $\phi_{k}\left(u,v\right)$ are phase spectra of the original SAR image and convolution kernel, respectively. $Corrcoef\left[\cdot \right]$ represents the process of obtaining the correlation coefficient. Empirical studies confirm that edge feature extraction significantly enhances SAR-based target discrimination. Leveraging this principle, TDP-SAR implements adaptive kernel pruning during recognition phases, selectively disregarding geometric descriptors to optimize computational efficiency. The framework establishes a quantitative validation protocol utilizing identical target signatures, where phase correlation metrics between original and convolved reconstructions serve as architectural optimization criteria. The pruning threshold, i.e., *th_c_*, can be confirmed by the Neyman–Pearson lemma.

## 4. Experiments and Discussions

In this section, we analyze the performance of TDF-SAR. This includes an adaptability analysis of multiple SAR target recognition models for SAR images of varying quality prior to pruning, as well as amplitude and phase spectra analysis of convolutional kernels. Finally, we perform a performance comparison before and after pruning. This demonstrates that TDF-SAR not only achieves model lightweighting but also maintains strong adaptability.

### 4.1. Experimental Setup

(1) Dataset

To validate the effectiveness of the proposed method, our experiments employed the MSTAR database, which is jointly funded by the Defense Advanced Research Projects Agency (DARPA) and the Air Force Research Laboratory (ADRL). The MSTAR database contains 10-class SAR target images with 0.3 m × 0.3 m resolution. Most SAR images in the dataset are sized 128 px × 128 px. To standardize input dimensions, we followed Reference [4] to crop original SAR images to 88 px × 88 px, which served as the ground truth for subsequent experiments. Training and test datasets were constructed using SAR images with 17° and 15° pitch angles, respectively, containing 2746 and 2425 samples each.

To validate adaptability to speckle noise, we augmented original training samples with Gamma-distributed simulated speckle noise (*L* = 1, 2, 5) to create a synthetic training dataset with varying quality levels. During testing, we evaluated samples containing Gamma-distributed noise across 11 noise levels (*L* = 0.2, 0.5, 1, 1.5, 2, 3, 5, 10, 20, 40, and 80).

(2) Experimental details

The following are the configurations used by the experimental server. Intel Core i7 CPU (Intel, Santa Clara, CA, USA), Ubuntu 20.04 operating system, and NVIDIA RTX 3090 Ti GPU (NVIDIA, Santa Clara, CA, USA) were used in the experiment. In order to train, we used the PyTorch 1.12.0 deep learning framework with CUDA 11.7, which was used with the Adam optimizer for the following parameters: weight decay of one by ten, batch size of 64, learning rate of one and a half, and initial stopping of 500 training epochs.

### 4.2. Comparison of SAR Target Recognition in Different Models

**Figure 5:**
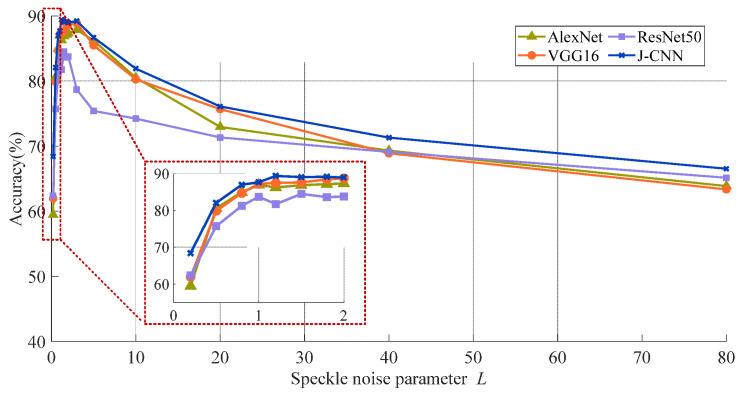
The adaptability comparison to SAR image quality in four models.

Section 2 highlights that in SAR images, the amount of natural speckle noise lowers the quality of the image and the degree of intensity in real world situations, but it varies. As such, we examine the effect of speckling noise in SAR target recognition models based on the CNN. Four SAR target identification models are trained and assessed. Figure 5 depicts recognition accuracies at various noise levels; the speckle noise level (*L*) is represented by the *x*-axis. (Higher *L* values point to worse SAR image quality.) Higher values of the image show a worse level of SAR.

**Figure 6:**
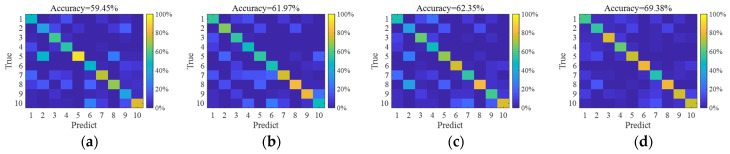
The confusion matrixes of four CNN models for SAR images with *L* = 0.2. (**a**) AlexNet, (**b**) VGG16, (**c**) ResNet50, and (**d**) J-CNN.

As described in the dataset section, model training samples consist of SAR images with simulated speckle noise at *L* = 1, 2, and 5. The accuracies are notably higher when test noise levels approximate these training values (*L* ≈ 1, 2, and 5). Even with high-quality SAR images (large *L* values), accuracy decreases significantly. This indicates that model performance degrades when testing conditions deviate from training parameters, revealing an overfitting problem in SAR target recognition. Nevertheless, J-CNN demonstrates superior adaptability to strong speckle noise compared to AlexNet, VGG16, and ResNet50. Particularly under *L* < 2 conditions (highlighted results), the accuracy of J-CNN becomes pronounced. Confusion matrices for all four CNN models with *L* = 0.2 are shown in Figure 6, where J-CNN achieves the highest accuracy (69.38%), reaffirming its exceptional noise robustness.

### 4.3. Analysis of Amplitude Spectrum in Convolution Kernel

According to Figure 5 results, J-CNN demonstrates the strongest adaptability to SAR images with severe speckle noise compared to classical CNN models. In this section, we analyze the reasons for this superior performance using the methodology described in Section 3.

**Figure 7:**
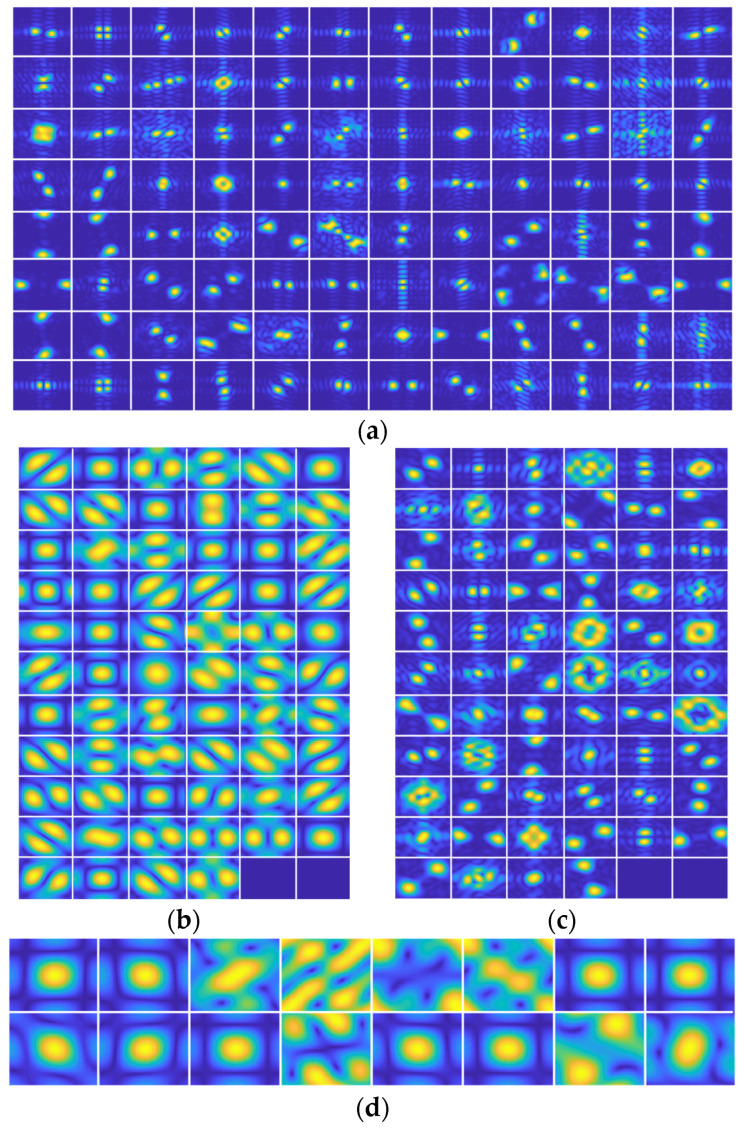
The amplitude spectrum of kernels in the first convolution layer. (**a**) AlexNet, (**b**) VGG16, (**c**) ResNet50, and (**d**) J-CNN.

Figure 7 displays the amplitude spectrum of all convolutional kernels in the first layer across the four networks, standardized to 128 px ×128 px. Yellow and blue regions denote high- and low-energy-spectrum components, respectively. Granular artifacts in SAR images, i.e., speckle noise, predominantly reside in high-frequency ranges. Crucially, only 4 of the 16 kernels in Figure 7d suppress low-band signals while emphasizing high-band feature extraction. Compared with this case, the amplitude spectrum of the first convolution layer in AlexNet and ResNet50 shown in Figure 7a,c has a significantly smaller area of a high-energy region. Additionally, the extracted frequency range information illustrates the strong pertinence. However, for SAR images with strong speckle noise, strong pertinence may lose the overall information of the SAR target but highlight the expression of granular noise. Moreover, in Figure 7b, the amplitude spectrum of the first convolution layer of VGG16 has a large area of high energy, but the retention of low-frequency band information is obviously less than J-CNN. It will lose the information on target contour and shape information in SAR images.

**Figure 8:**
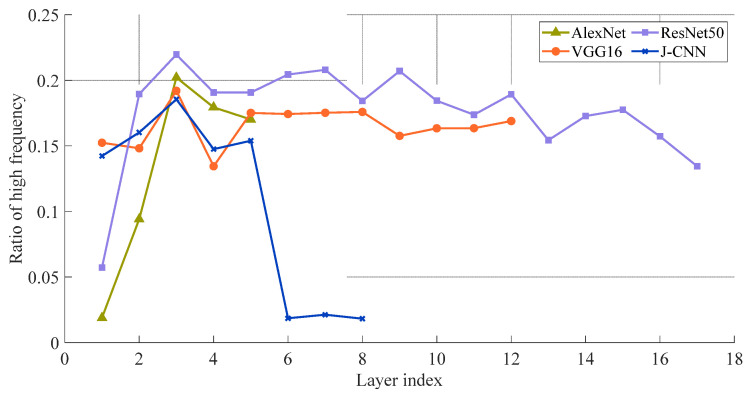
The high frequency ratio layer by layer.

**Figure 9:**
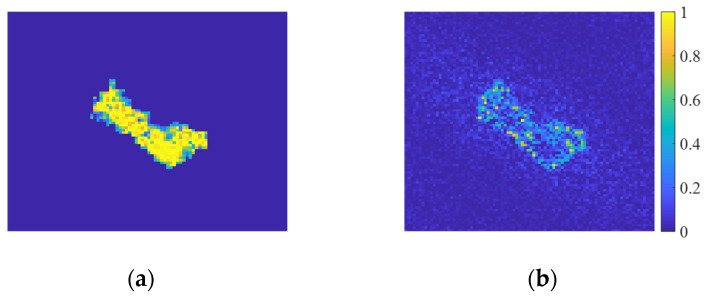
SAR target image and its reconstruction result. (**a**) SAR target image with zeroed clutter; (**b**) reconstruction result based on phase spectrum.

For a quantitative comparison of convolutional kernel amplitude spectra across the four models, Figure 8 layers the high frequency ratios using Equation (13), with hyperparameters *th_r_* = 0.7 and *R* = 70. The axes denote convolutional layer indices (horizontal) and high frequency ratios (vertical), excluding all 1 × 1 convolutional layers in this analysis. As shown in Figure 9, the J-CNN maintains elevated high frequency ratios across its first five convolutional layers, followed by a marked decline starting from the sixth layer. This aligns with J-CNN’s hierarchical design: its first five layers perform speckle reduction, while the final three layers constitute the classification stage. This structural dichotomy demonstrates that J-CNN’s speckle reduction phase actively preserves SAR target details, whereas the classification stage leverages generalized features for target identification, achieving balanced optimization of both objectives. Comparatively, AlexNet and ResNet50 prioritize general target information extraction in shallow layers (notably the first convolutional layer) while reserving detailed feature analysis for deeper layers. For SAR images affected by strong speckle noise, the low convolutional layer will extract more general speckle noise. Then, detailed information of speckle noise will be paid attention to in the deep layers. As a result, the adaptability is poor enough such that these models’ SAR images are affected by strong speckle noise. The high frequency ratio of VGG16 has little change with the deepening of the convolutional layers and always maintains a high level. It shows that VGG16 always pays attention to the extraction of detailed features in the high-frequency band. In the case of strong speckle noise, the details will affect the recognition results. Based on this, the reasons for the strong adaptability of the J-CNN to speckle noise are analyzed from the perspective of the convolution kernel amplitude spectrum.

### 4.4. Analysis of Phase Spectrum in Convolution Kernel

This section analyzes J-CNN’s exceptional noise adaptability through phase spectrum characteristics. A SAR target image with zeroed clutter regions (Figure 9a) serves as the analytical basis. Reconstruction via Equation (15) yields the output in Figure 9b, where the phase spectrum distinctly encodes target morphology and positional data.

**Figure 10:**
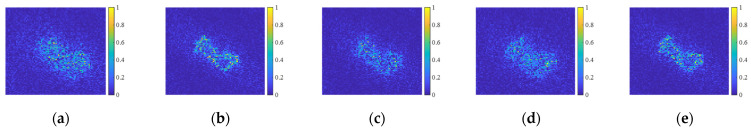
The reconstruction results based on the phase spectrum. (**a**) AlexNet, (**b**) VGG16, (**c**) ResNet50, (**d**) first layer of the J-CNN, and (**e**) the sixth layer of the J-CNN.

We select convolutional kernels from the first layers of the AlexNet, VGG16, ResNet50, and J-CNN, along with the sixth layer of the J-CNN. Reconstruction results generated via Equation (17) are shown in Figure 10. The phase spectrum of VGG16’s first layer and J-CNN’s sixth layer exhibit superior contour preservation, whereas other models lack this capability. Notably, J-CNN’s first layer prioritizes target-generalized features over shape-specific details, explaining its enhanced adaptability to SAR images of varying quality. Specifically, the J-CNN emphasizes holistic image information during speckle reduction while suppressing positional/shape details. These features are selectively reactivated only in later recognition stages.

**Figure 11:**
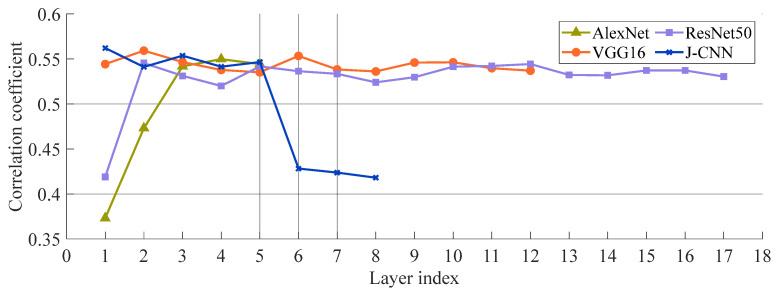
The mean value of correlation coefficients in every layer.

We further quantify the phase spectrum of convolutional kernels using correlation coefficients derived from Equation (18). Figure 11 displays the layer-wise correlation coefficients for all four models. Notably, the J-CNN exhibits marked spectrum divergence between its despeckling and recognition stages. During despeckling, its convolutional phase spectrum exerts minimal influence on target geometry—a strategic suppression of shape and positional encoding. This design intentionally avoids extracting spurious spatial features from speckle-dominated regions, where noise artifacts frequently mimic target characteristics. However, there is a considerable increase in the correlation coefficient in the recognition stage, which shows that the target position and shape encoding are given more attention. AlexNet and ResNet50, some of the well-established models, show reduced correlation coefficients in shallow layers, which suggests that there is a greater influence of the phase spectrum at early processing stages. While deeper layers move toward generalized representations for subsequent tasks, these layers place a high value on the extraction of positional and shape features. Because of this architecture, SAR imagery may be able to propagate speckle-induced artifacts because low-level convolutions might misunderstand granular noise because of structural characteristics. VGG16, on the other hand, maintains somewhat high correlation coefficients throughout layers, which indicates that SAR target geometry reconstruction has a limited amount of dependence on the phase spectrum.

### 4.5. Comparison of Model Performance Pre- and Post-Prunning

The proposed method employs the J-CNN as its baseline model, explicitly decoupling despeckling and recognition stages. We implement pruning via TDP-SAR (detailed in Section 3) to validate its effectiveness. Ablation experiments compare pruned model accuracies across three configurations as follows: amplitude spectrum-only pruning, phase spectrum-only pruning, and full TDP-SAR. Results in Table 3 evaluate the performance on SAR images with varying noise levels (*L* = 0.2, 0.5, 1, 2, 3, and 5), including accuracy drop ratios between TDP-SAR and the original J-CNN. Threshold hyperparameters for TDP-SAR are set as *th_r_* = 0.7 and *th_c_* = 0.5 by the Neyman–Pearson lemma.

As shown in Table 3, the J-CNN achieves the highest recognition accuracy across most SAR image quality levels. When pruned via the amplitude spectrum, phase spectrum, or TDP-SAR strategies, its accuracy exhibits only marginal declines—with all degradation rates remaining below 1%, a practically acceptable threshold. Notably, at specific noise levels (*L* = 1 and *L* = 3), the pruned models retain or even slightly surpass baseline accuracy. These results confirm that TDP-SAR introduces minimal degradation to J-CNN’s recognition performance while significantly reducing model complexity, thereby enabling effective compression.

**Figure 12:**
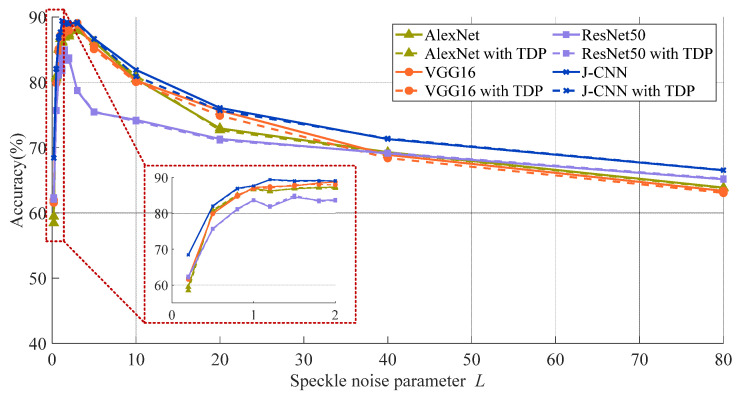
Comparison of recognition accuracy of models before and after pruning.

We further apply TDP-SAR pruning to the AlexNet, VGG16, ResNet50, and J-CNN. Figure 12 compares their recognition accuracies pre- and post-pruning, where solid lines denote baseline performance and dashed lines represent pruned results. For classical CNNs without dedicated despeckling stages (AlexNet, VGG16, ResNet50), phase spectrum-based pruning is directly implemented. The near-overlapping curves in Figure 12 demonstrate minimal accuracy variation across all pruned models, confirming that TDP-SAR preserves recognition capability while achieving parameter reduction.

We quantified parameter counts and inference times for some classical models pre- and post-pruning, such as the AlexNet, VGG16, ResNet50, EfficientNet-B0 [33], and MobileNet [34], with results summarized in Table 4. Pruning reduces parameter volumes significantly across all four SAR recognition networks. Most notably, the J-CNN achieves a 17.7% parameter reduction relative to its original size, demonstrating the effectiveness of TDP-SAR in model lightweighting. Execution times also decrease measurably for all pruned networks, further validating the practical utility of the strategy for deployment scenarios.

### 4.6. Comparison of Prunning Methods

To further validate the pruning performance of TDP-SAR, we conducted comparative experiments with different pruning methods, including L1 norm [35], HRank [36], and FiltDivNet [37]. The results are presented in Table 5. All baseline models employed the J-CNN, with test data comprising synthetic SAR images containing simulated noise at *L* = 5. Table 5 demonstrates the variations in model recognition accuracy and the number of weight parameters before and after applying different pruning methods. Comparative analysis reveals that while our method does not achieve the most significant parameter reduction, it maintains recognition accuracy after pruning due to its focus on retaining effective features extracted at different processing stages. In contrast, other classical pruning methods, particularly FiltDivNet, demonstrate optimal parameter reduction but exhibit reduced adaptability to SAR images under strong noise interference, resulting in a 2% degradation in recognition accuracy post-pruning.

## 5. Conclusions

A TDP strategy is presented in this paper for SAR target recognition CNN models. In contrast to traditional pruning approaches, which do away with characteristics based on generic importance measures, TDP-SAR uses different target identification stages to analyze the convolutional kernel spectrum. In the despeckling phase, convolutional kernels with the dominating high-frequency amplitude spectrum are the main focus of pruning. Optimization in the recognition phase is focused on maintaining the features of the phase spectrum. High-correlation-coefficient convolutional kernels are eliminated, which shows that the phase spectrum of convolutional kernels have little effect on recognition. Experiments employ the classical MSTAR dataset. Recognition result comparisons first demonstrate the superior SAR speckle noise adaptability of the proposed J-CNN. Notably, at *L* = 0.2, the J-CNN achieves higher accuracy than classical CNNs (AlexNet, VGG16, ResNet50). Frequency domain analysis further explains this robustness: the despeckling stage of the J-CNN preserves SAR target details through amplitude spectrum optimization, while its recognition stage leverages generalizable phase spectrum features. Both despeckling and recognition performance must be jointly optimized. In the despeckling stage of the J-CNN, convolutional kernels exhibit minimal phase spectrum influence—intentionally disregarding target shape and positional information to mitigate speckle noise interference. Conversely, the recognition stage prioritizes these geometric features. Post-pruning comparisons reveal no significant accuracy degradation despite substantial parameter reductions. Here, we quantified parameter counts and inference times for some classical models pre- and post-pruning. The J-CNN achieves a 17.7% parameter reduction relative to its original size, demonstrating the effectiveness of TDP-SAR in model lightweighting. Notably, the J-CNN retains robust adaptability to varying SAR image quality levels even under TDP-SAR-based pruning. This work advances the practical deployment of deep learning models for SAR target recognition. In future work, we will further apply the TDP-SAR strategy to the transfer learning task for SAR images to obtain a more efficient SAR image processing model.

## Figures and Tables

**Table 1:** Convolution kernel settings in convolution layers of J-CNN.

Index of Convolution Layer	J-CNN (A@B × B)
1	16@3 × 3
2	256@3 × 3
3	256@3 × 3
4	256@3 × 3
5	16@3 × 3
6	6@9 × 9
7	36@9 × 9
8	36@9 × 9

**Table 2:** Comparison of CNN models.

CNN Models		Numbers of Weights	Achievement
Classical models	AlexNet	60 M	Champion of the ImageNet Challenge in 2012
	VGG16	138 M	Proposed by the Visual Geometry Group of the University of Oxford in 2014
	ResNet50	26 M	Champion of the ImageNet Challenge in 2015
J-CNN		26,746	-

**Table 3:** Ablation experiment results.

L	Accuracy (%)				Accuracy Drop Ratio (%)
	J-CNN	Amplitude Spectrum-Only	Phase Spectrum-Only	TDP-SAR	
0.2	68.41	68.24	68.44	68.39	0.3
0.5	82.06	81.88	81.91	81.49	0.7
1	87.67	87.71	87.66	87.67	-
2	89.15	89.03	89.12	89.1	0.6
3	88.95	88.9	88.75	88.98	−0.3
5	86.68	86.15	86.19	86.61	0.8

**Table 4:** Comparison of network complexity.

Network	With Pruning	Numbers of Weights	Percentage Reduction in Parameters (%)	Execution Time (ms)
AlexNet	×	60 M	7.7	7.18
	√	55.4 M		6.59
VGG16	×	138 M	16.7	8.58
	√	115 M		7.2
ResNet50	×	26 M	15.4	3.01
	√	22 M		2.53
EfficientNet-B0	×	5.3 M	3.8	1.22
	√	5.1 M		1.17
MobileNet	×	4.3 M	7	1.11
	√	4.0 M		0.99
J-CNN	×	26,746	17.7	0.87
	√	22,051		0.7

Note: × is without pruning operation, √ is with pruning operation.

**Table 5:** Comparison with other pruning methods for J-CNN on synthetic SAR images with *L* = 5 simulated speckle noise.

Pruning Method	Accuracy (%)		Number of Weights		
	Baseline Model	Pruned Model	Baseline Model	Pruned Model	Percentage Reduction in Parameters (%)
L1 norm [35]	86.68	85.55	26,746	23,082	13.70
HRank [36]		85.60		20,771	22.34
FiltDivNet [37]		84.65		20,104	24.83
TDP-SAR (Ours)		86.61		22,051	17.55

## Data Availability

The original contributions presented in this study are included in the article. Further inquiries can be directed to the corresponding author.
